# Author Correction: Nuclear RPSA senses viral nucleic acids to promote the innate inflammatory response

**DOI:** 10.1038/s41467-024-45275-2

**Published:** 2024-01-29

**Authors:** Yan Jiang, Siqi Sun, Yuan Quan, Xin Wang, Yuling You, Xiao Zhang, Yue Zhang, Yin Liu, Bingjing Wang, Henan Xu, Xuetao Cao

**Affiliations:** 1 Department of Immunology, Center for Immunotherapy, Institute of Basic Medical Sciences, Peking Union Medical College, Chinese Academy of Medical Sciences, Beijing, 100005 China; 2 Frontiers Science Center for Cell Responses, Institute of Immunology, College of Life Sciences, Nankai University, Tianjin, 300071 China

**Keywords:** Innate immunity, Acute inflammation

Correction to: *Nature Communications* 10.1038/s41467-023-43784-0, published online 20 December 2023

The original version of this Article contained an error in Figure 6.

In the original version of Fig. 6, the incorrect control of PBS treated lung tissue from Rpsa fl/fl Lyz-Cre+ mice was shown in panel i. The correct version of Fig. 6 is:
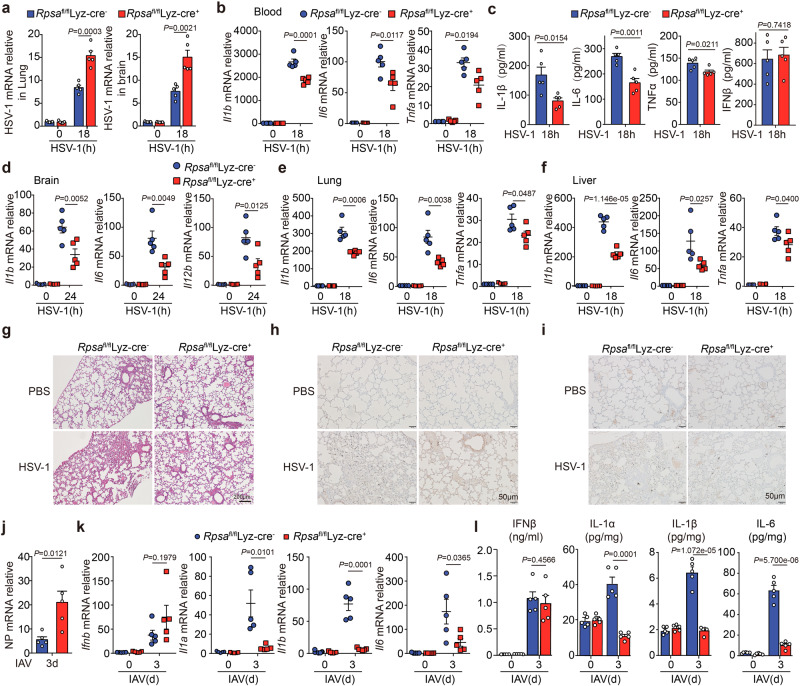
which replaces the previous incorrect version:
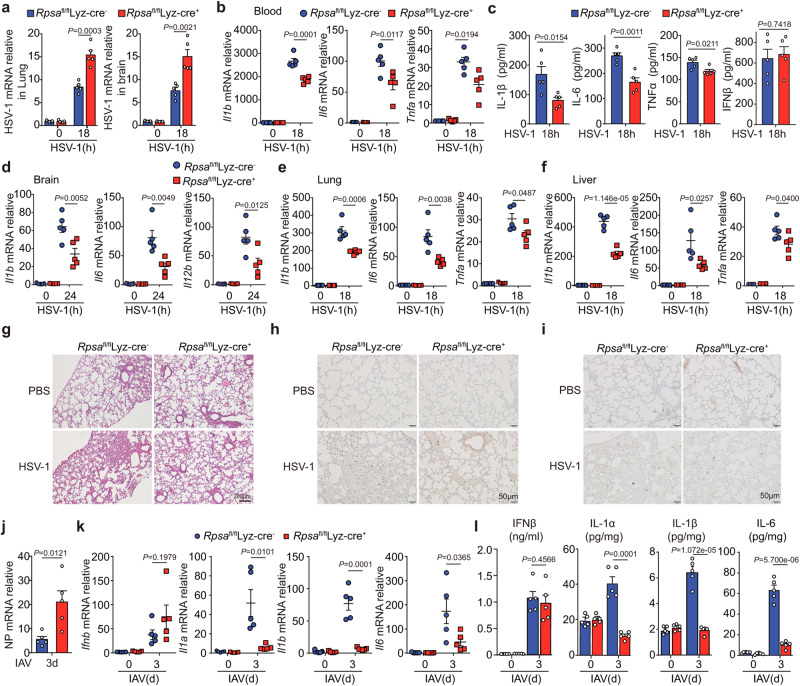


This has been corrected in both the PDF and HTML versions of the Article.

The original version of this Article contained an error in the Source Data for Figure 6i.

The Source Data for Figure 6i originally showed the incorrect control panel of PBS treated lung tissue from Rpsa fl/fl Lyz-Cre+ mice. The correct version replaces this panel with the correct microscopy image.

The HTML has been updated to include a corrected version of the Source Data.

### Supplementary information

Corrected Source Data

