# Supplementary material for: Nuclear RPSA senses viral nucleic acids to promote the innate inflammatory response
Source: Nat Commun. 2023 Dec 20;14:8455. doi: 10.1038/s41467-023-43784-0 (PMC10730619; doi:10.1038/s41467-023-43784-0)
Supplement: Supplementary file 1 — Supplementary information [file 41467_2023_43784_MOESM1_ESM.pdf]

Supplementary information

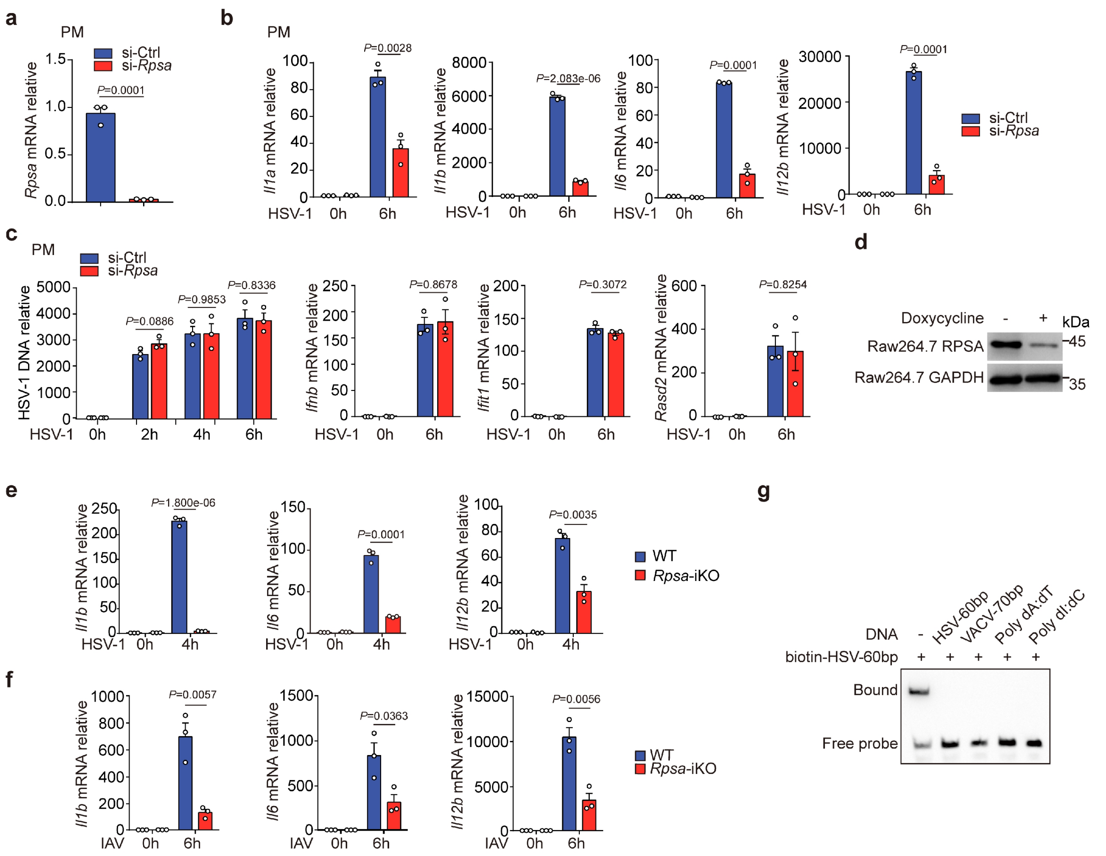

Supplementary Figure 1. RPSA promotes HSV-1 infection-induced proinflammatory cytokine gene expression and binds pathogenic dsDNA

a-c Mouse PMs were transfected with control siRNA or *Rpsa*-specific siRNA for 48 h then infected with HSV-1 for the indicated time. *Rpsa* mRNA (**a**), *Il1a, Il1b, Il6, Il12b* mRNA (**b**) *Ifnb*, *Ifit1*, *Rasd2* mRNA and HSV-1 DNA (**c**) levels were examined by qRT-PCR (n = 3). **d** Confirmation of inducible knockout of RPSA in RAW264.7 cell line by immunoblot. **e-f** Wild-type and *Rpsa*-iKO RAW264.7 cells were infected with HSV-1(MOI,10) (**e**) or IAV (MOI,1) (**f**) for the indicated time, and then the levels of pro-inflammatory cytokine mRNAs were determined by qRT-PCR (n = 3). **g** EMSA analysis of recombinant mouse RPSA (rmRPSA) binding to the indicated DNA substrates. Similar results were obtained for three independent experiments. One

representative experiment is shown. Data in **a-c, e** and **f** are shown as mean ± s.e.m.. The *P* values were calculated by a two-tailed unpaired Student's *t*-test. Source data are provided as a Source Data file.

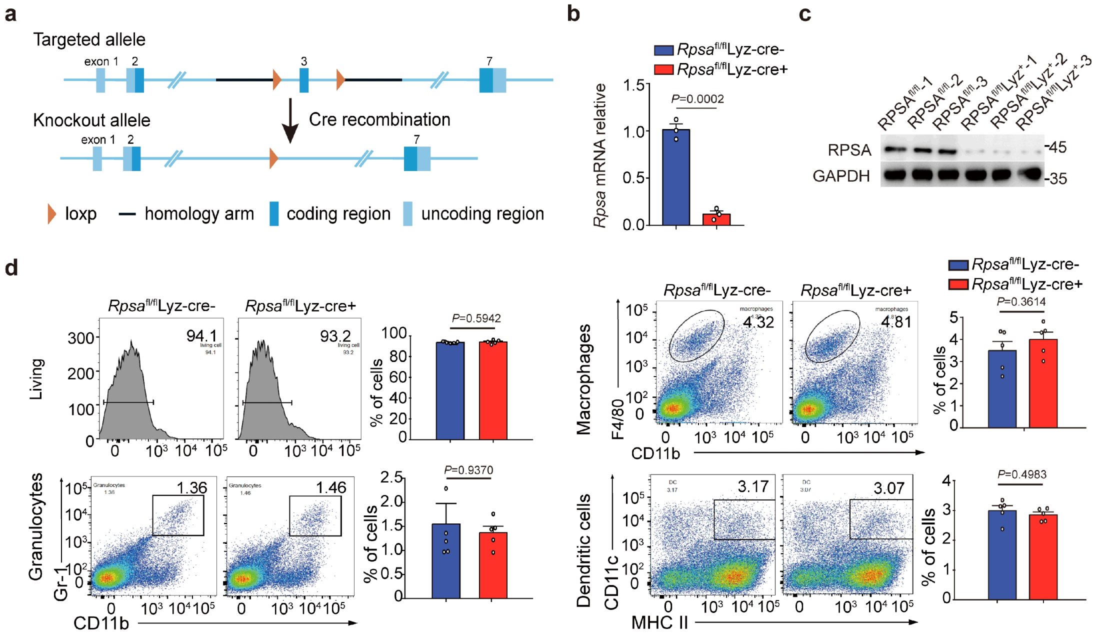

Supplementary Figure 2. Loss of *Rpsa* does not disturb the development of immune cells *in vivo*

a Schematic diagram of *Rpsa*$^{fl/fl}$ mouse design strategy and generation of conditional *Rpsa* deficient mice. **b** qRT-PCR analysis of the *Rpsa* expression in BMDMs of *Rpsa*$^{fl/fl}$ Lyz-Cre+ mice and the littermates (n = 3). **c** The protein levels of RPSA in BMDMs were determined by immunoblot. **d** Representative FACS profiles of splenocytes prepared from *Rpsa*$^{fl/fl}$ Lyz-Cre+ mice and the littermates (n=5). Cells were stained with indicated fluorochrome-conjugated antibodies. FACS profiles were scatter-gated on live cells. Gated cell populations include CD11b$^+$F4/80$^+$ macrophages, CD11b$^+$Gr-1$^+$ granulocytes and CD11c$^+$MHC-II$^+$ dendritic cells. Similar results were obtained for two independent experiments and one representative experiment is shown. Data in **b**, **d** are shown as mean ± s.e.m.. The *P* values were calculated by a two-tailed unpaired Student's *t*-test with confidence interval of 95%. Source data are provided as a Source Data file.

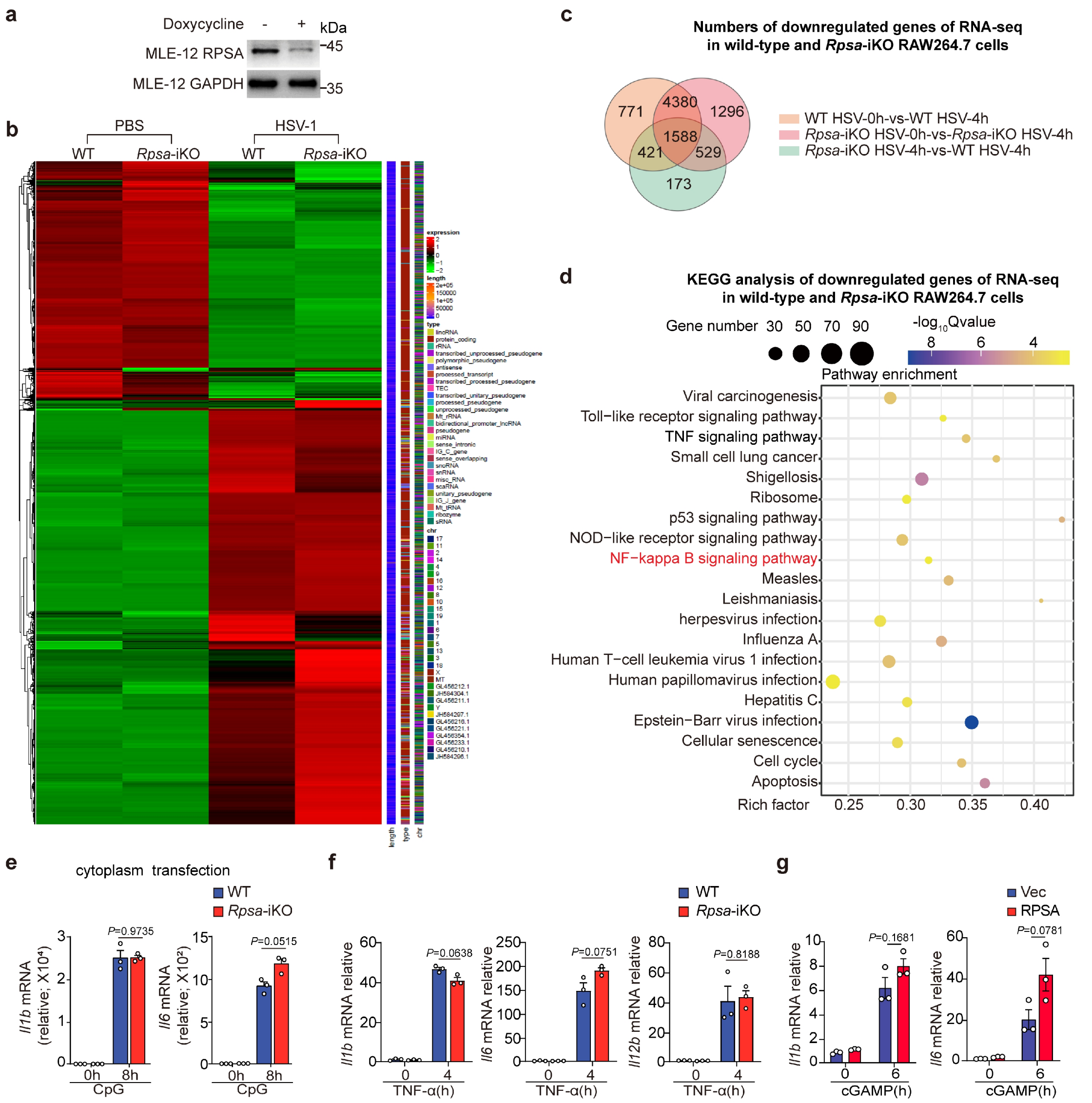

Supplementary Figure 3. Deficiency of *Rpsa* inhibits the expression of proinflammatory cytokines in response to viral infection

a Confirmation of inducible knockout of RPSA in MLE-12 cell line by immunoblot. **b-d** Bioinformatics analysis of RNA-seq in wild-type and *Rpsa*-iKO RAW264.7 cells in response to HSV-1 infection. **b** Heatmap of global clustering of gene expression in *Rpsa*-iKO and wild-type RAW264.7 cells in response to HSV-1 infection. Statistics (**c**) and KEGG analysis (**d**) of downregulated genes in data from **b**. **e** qRT-PCR analysis of *Il1b and Il6* mRNAs in wild-type and *Rpsa*-iKO RAW264.7 cells lipidosome

transfected with 5μM CpG ODNs for 8h (n = 3). **f** Wild-type and *Rpsa*-iKO RAW264.7 cells were stimulated with Tnf-α (10ng/ml) for 4h and then the levels of pro-inflammatory cytokine mRNAs were determined by qRT-PCR (n = 3). **g** qRT-PCR analysis of *Il1b and Il6* mRNAs in wild-type and *Rpsa*-iKO RAW264.7 cells stimulated with 5μg/ml cGAMP for 6h (n = 3). Similar results were obtained for three independent experiments and one representative experiment is shown (a, e-g). Data in **e**-g are shown as mean ± s.e.m.. The *P* values were calculated by a two-tailed unpaired Student's *t*-test with confidence interval of 95%. Source data are provided as a Source Data file.

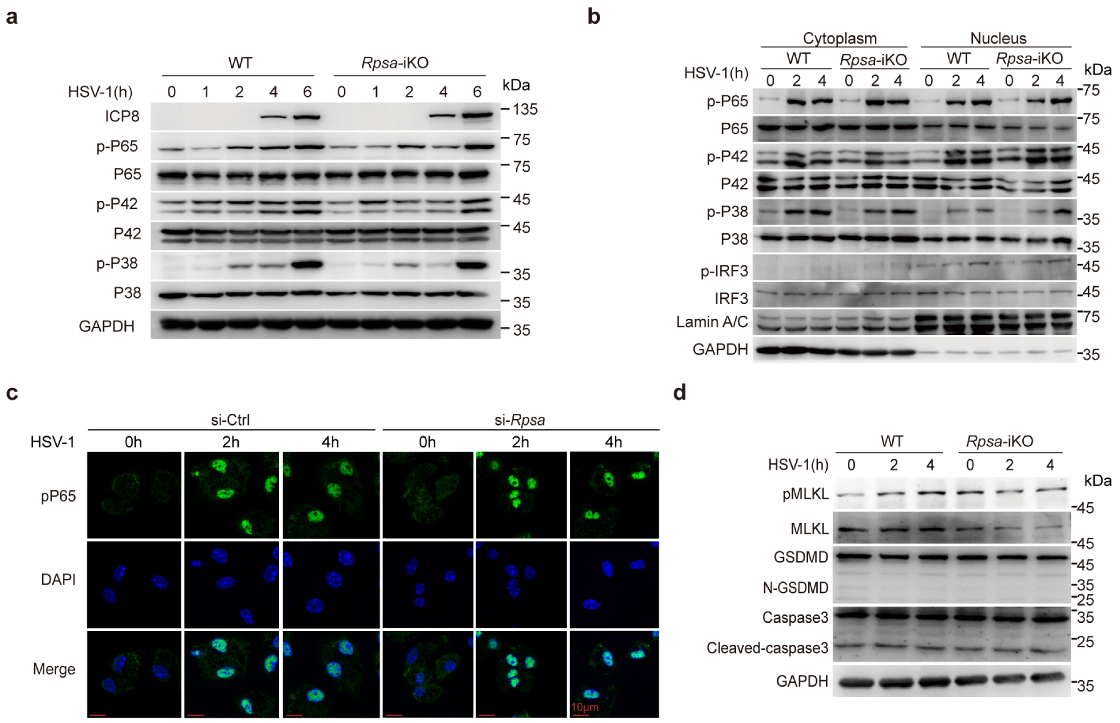

Supplementary Figure 4. RPSA promotes proinflammatory cytokine gene transcription without affecting innate signaling and death pathways

a Immunoblot analysis for p- and total P65, P42, and P38 as well as GAPDH in wild-type and *Rpsa*-iKO RAW264.7 cells infected with HSV-1 for the indicated time. **b** Distribution of indicated proteins in cytoplasmic and nuclear of Wild-type and *Rpsa*-iKO RAW264.7 cells infected with HSV-1 were determined by immunoblot analysis. **c** Distribution of P65 (green) in BMDMs without or with HSV-1 infection was examined by immunofluorescence assay. Nuclei were stained with DAPI (blue). Scale bar=10 μm. **d** Immunoblot analysis of p- and total MLKL, GSDMD/N-GSDMD, caspase3/cleaved-caspase3 and GAPDH in wild-type and *Rpsa*-iKO RAW264.7 cells infected with HSV-1. Similar results were obtained for three independent experiments and one representative experiment is shown. Source data are provided as a Source Data file.

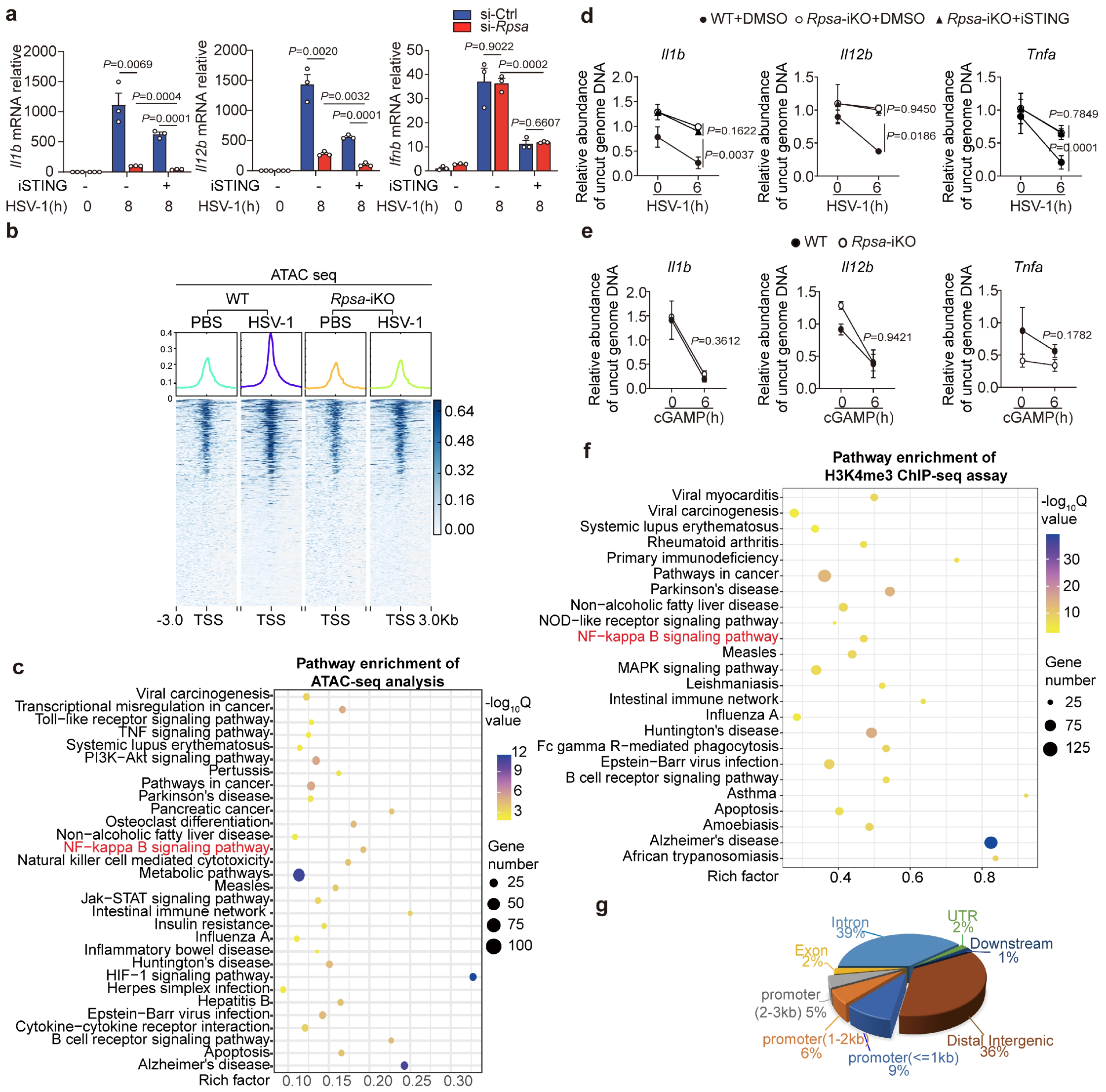

Supplementary Figure 5. RPSA promotes proinflammatory cytokine expression by enhancing chromatin accessibility.

a. Mouse PMs were transfected with control siRNA or *Rpsa*-specific siRNA for 48 h and then pretreated with or without the STING inhibitor (C-178 5μM) for 2h, before being infected with or without HSV-1 for 8h. *Il1b*, *Il12b* and *Ifnb* mRNAs and examined by qRT-PCR (n = 3). **b** Distribution of reads relative to TSS from ATAC-seq of wild-type and *Rpsa*-iKO RAW264.7 cells infected with HSV-1 for 4 hours. **c** KEGG pathway analysis of genes associated with differential peaks from ATAC-seq of wild-type and *Rpsa*-iKO RAW264.7 cells infected with HSV-1 for 4h. **d** DNase I sensitivity assay at

promoter regions of *Il1b, Il12b,* and *Tnfa* in wild-type and *Rpsa*-iKO RAW264.7 cells pretreated with or without the STING inhibitor (C-178 5μM) for 2h, and then infected with or without HSV-1 for 6h (n = 3). **e** DNase I sensitivity assay at promoter regions of *Il1b, Il12b,* and *Tnfa* in wild-type and *Rpsa*-iKO RAW264.7 cells infected with cGAMP (5μg/ml) (n = 3). **f** KEGG pathway analysis of genes associated with differential peaks from H3K4me3 ChIP-seq of wild-type and *Rpsa*-iKO RAW264.7 cells infected with HSV-1 for 4h. **g** Regional distribution of differential peaks on chromatin was shown. Similar results were obtained for three independent experiments and one representative experiment is shown (a, d and e). Data in **a, d** and **e** are shown as mean ± s.e.m., The *P* values were calculated by a two-tailed unpaired Student's *t*-test with confidence interval of 95%. Source data are provided as a Source Data file.

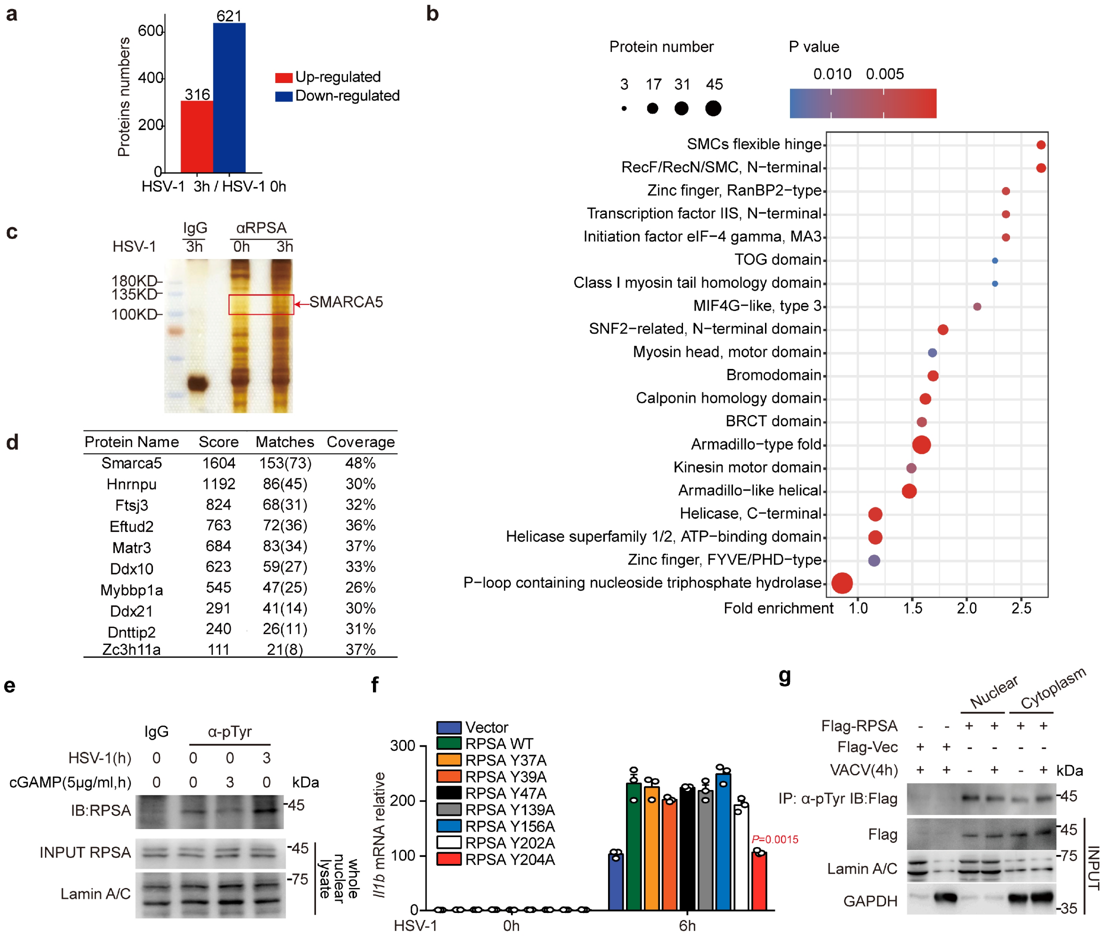

Supplementary Figure 6. RPSA interacts with SMARCA5 in macrophages upon HSV-1 infection.

a Numbers of differential RPSA-interacting proteins in HSV-1 infected BMDMs identified by non-targeted quantitative mass spectrometry. **b** Enrichment of up-regulated RPSA-interacting protein domains in BMDMs after HSV-1 infection. **c-d** Silver-stained protein gel analyzed for RPSA-interacting proteins, differential immunoprecipitated protein band indicated by red arrows and red rectangles (**c**) and the top 10 overlapped candidates were listed in (**d**). **e** Nuclear extracts from RAW264.7 cells infected with HSV-1 or stimulated with cGAMP(5μg/ml) were immunoprecipitated with anti-Phospho-Tyrosine antibody (α-pTyr) or IgG then

immunoblotted for RPSA (n = 3). **f** qRT-PCR analysis for *Il1b* mRNA levels in wild-type and *Rpsa*-iKO MLE-12 cells transfected with the indicated plasmids then infected with HSV-1. **g** Cell lysates from HEK293T cells, which were transfected with the indicated plasmids and then infected with VACV, were immunoprecipitated with anti-Phospho-Tyrosine antibody (α-Tyr). Then RPSA was detected through immunoblot analysis. Similar results were obtained for three independent experiments and one representative experiment is shown (e-g). Data in **f** are shown as mean ± s.e.m.. The *P* values were calculated by a two-tailed unpaired Student's *t*-test with confidence interval of 95%. Source data are provided as a Source Data file.

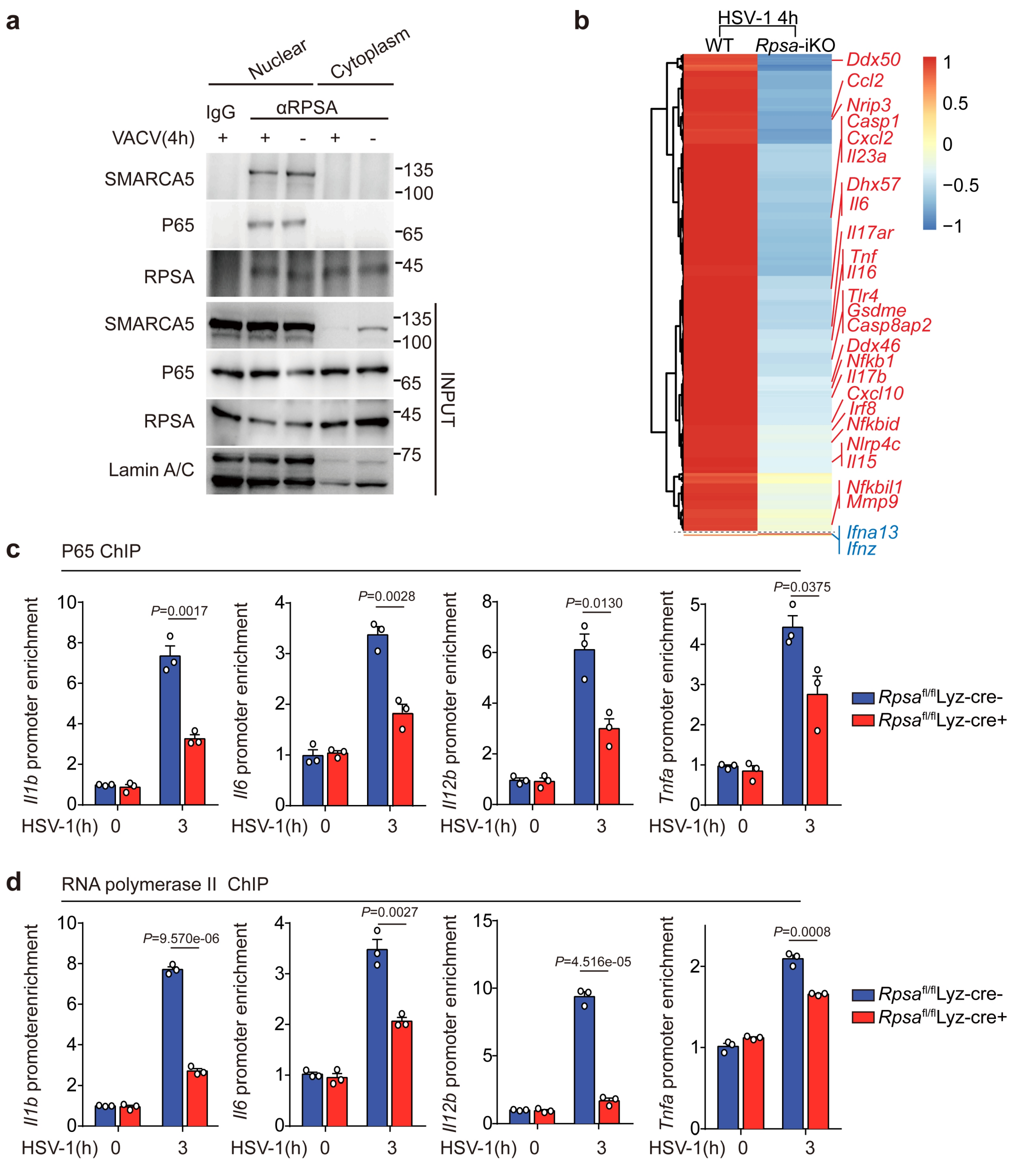

Supplementary Figure 7. RPSA is required for p65-mediated proinflammatory cytokine gene transcription.

a Cell lysates from RAW264.7 cells infected with VACV were immunoprecipitated with anti-RPSA antibody or IgG then immunoblotted for indicated proteins. **b** Clustering of downregulated enriched peaks of P65 ChIP-seq analysis in *Rpsa*-iKO RAW264.7 cells relative to wild-type RAW264.7 cells after HSV-1 infection. **c-d** ChIP assay of P65 subunit (c) and RNA polymerase II (d) recruitment to the *Il1b*, *Il6*, *Il12b* and *Tnfa* promoter sites in BMDMs from *Rpsa*$^{fl/fl}$ Lyz-Cre+ mice and littermates (n = 3).

Similar results were obtained from three independent experiments and one representative experiment is shown (**a**, **c** and **d**). Data in **c** and **d** are shown as mean ± s.e.m.. The *P* values were calculated by a two-tailed unpaired Student's *t*-test with confidence interval of 95%. Source data are provided as a Source Data file.

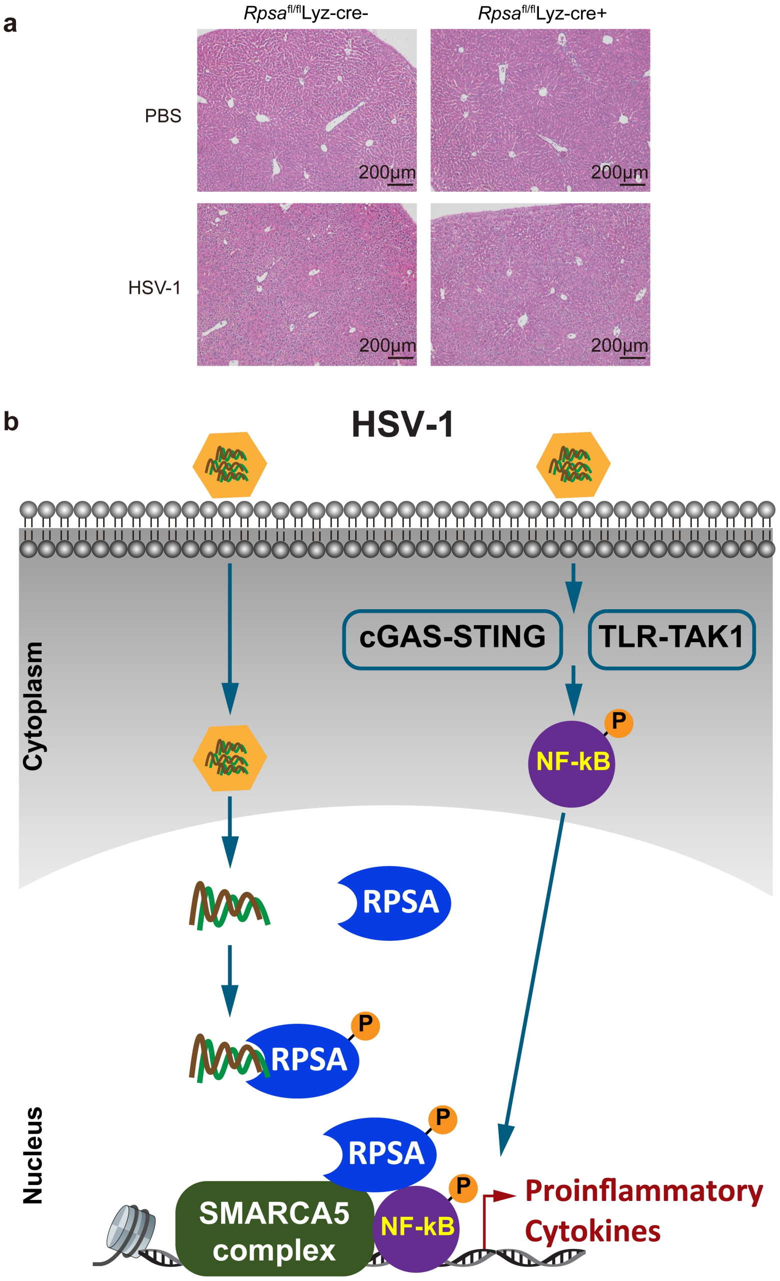

Supplementary Figure 8. Less inflammatory damage in the liver of *Rpsa*$^{fl/fl}$ Lyz-Cre+ mice after HSV-1 infection

a *Rpsa*$^{fl/fl}$ or *Rpsa*$^{fl/fl}$ Lyz-Cre+ mice were inoculated intravenously (i.v.) with HSV-1 at 8×10^7 plaque-forming units (PFU) (n=5). HE staining of the liver. **b** Summative model of RPSA promoting transcription of pro-inflammatory cytokines in response to nuclear virus infection. Source data are provided as a Source Data file.

Table S1 Sequences for siRNA

Arpc2	TATCTGCACTACCACATTA
Cherp	GAGTTCTACAGTTACTACA
Fabp5	GAGAGCACGATAACAAGAA
Hnrnpa2b1	GGCTTAAGCTTTGAAACCA
Kif20b	CATCAGAACCAACGATCTA
Mpp1	GCAACATGTTTGGCACCAA
Ncl	GCCTTCTAAGGACATTCCA
Nxf1	GCGATGTAGCAATGAATGA
Pcbp2	ACTCACCATTCCAAATGAT
Pgam1	TCTATGAACTGGACAAGAA
Pkm	GATGTCGACCTTCGTGTAA
Pnpla2	CAGACAACTTGCCACTTTA
Psme3	GAACCAAGGTGTTTGTGAT
Rad51	GAAGCCGGTTACCATACAG
Rfc3	CAACTACCACCTTGAAGTTAA
Rpl23a	GCCAATAAGCATCAGATCA
Rps27a	CACGTGTGTGGCCGCCTAA
Rpsa	GCCATCCCATGCAACAACA
Shoc2	CTCAACTCACAGAACTTTA
Sod1	GGGCAAAGGTGGAAATGAA
Smarca5	GGGCAAATAGATTCGAGTA
scramble	TTCTCCGAACGTGTCACGT
Tpi1	AACTCATCTGCACCCTGAA
Ttn	CCACCATCGTTTATCAAGA

Ubc	TACCTTCCTCACCACAGTA

Table S2 SgRNA sequences for genes knock-out with Crispr-Cas9

Rpsa sgRNA-1 (sense)	A1	TTCTGCGGTTCGCGCCACGC
Rpsa sgRNA-1 (anti-sense)	A1	GCGTGGCGCGAACCGCAGAA
Rpsa sgRNA-2 (sense)	A2	TGCACACTATTAAAGCTCAG
Rpsa sgRNA-2 (anti-sense)	A2	CTGAGCTTTAATAGTGTGCA

Table S3 Primer sequences for qRT-PCR

Rpsa-F	GTGGCACCAACCTTGACTTTC
Rpsa -R	CAGCAGGATTCTCGATGGCA
Ifnb1-F	CAGCTCCAAGAAAGGACGAAC
Ifnb1-R	GGCAGTGTAACTCTTCTGCAT
Il1a-F	CGAAGACTACAGTTCTGCCATT
Il1a -R	GACGTTTCAGAGGTTCTCAGAG
Il1b-F	GCAACTGTTCCTGAACTCAACT
Il1b-R	ATCTTTTGGGGTCCGTCAACT
Il6-F	TAGTCCTTCCTACCCCAATTTCC
Il6-R	TTGGTGGTTTGTGAGTGTGAG
Tnfα-F	GACGTGGAACTGGCAGAAGAG
Tnfα-R	TGCCATTCTTGTTCGTGTAAGTT
Il12b -F	TGGTTTGCCATCGTTTTGCTG
Il12b -R	ACAGGTGAGGTTCACTGTTTCT
HSV-1-F	TCCGACAGCGATCTGGACGA
HSV-1-R	AGGCGCGACCACACACTGTG
IAV-P3-F	GGCCGACTACACTCTCGATGA
IAV-P3-R	TGTCTTATGGTGAATGACCTGGTTT
Il1b (NFκB site) -F	GTTCCGCACATCCTGACTTA
Il1b (NFκB site)-R	CAATTGTGCAGATGGTGTCAA
Il6 (NFκB site) -R	TTTCCAATCAGCCCCACC
Il6 (NFκB site) -R	CAGAATGAGCTACAGACATCCC
Il12b (NFκB site) -F	CATTTCCTCTTAACCTGGGATTTC
Il12b (NFκB site) -R	CTGCTCCTGGTGCTTATATACT
Tnfα (NFκB site)-F	CCAGCCAGCAGAAGCTCCCTCAGCGAG
Tnfα (NFκB site)-R	GCGGATCATGCTTTCTGTGCTCATGGTGTC

b-Actin-F	AGTGTGACGTTGACATCCGT
b-Actin -R	GCAGCTCAGTAACAGTCCGC
Gapdh-F	AGGTCGGTGTGAACGGATTTG
Gapdh -R	TGTAGACCATGTAGTTGAGGTCA
Ifit1-F	CTGAGATGTCACTTCACATGGAA
Ifit1-R	GTGCATCCCCAATGGGTTCT
Rsad2-F	TGCTGGCTGAGAATAGCATTAGG
Rsad2-R	GCTGAGTGCTGTTCCCATCT
hActinb-F	*CATGTACGTTGCTATCCAGGC*
hActinb-R	CTCCTTAATGTCACGCACGAT
hCXCL10-F	GTGGCATTCAAGGAGTACCTC
hCXCL10-R	TGATGGCCTTCGATTCTGGATT
hIFNB1-F	ATGACCAACAAGTGTCTCCTCC
hIFNB1-R	GGAATCCAAGCAAGTTGTAGCTC
hIL6-F	ACTCACCTCTTCAGAACGAATTG
hIL6-R	CCATCTTTGGAAGGTTCAGGTTG
hIL1B-F	ATGATGGCTTATTACAGTGGCAA
hIL1B-R	GTCGGAGATTCGTAGCTGGA
hIL1A-F	TGGTAGTAGCAACCAACGGGA
hIL1A-R	ACTTTGATTGAGGGCGTCATTC

Table S4 Reagents

REAGENT or RESOURCE	SOURCE	IDENTIFIER
Antibodies		
DYKDDDDK Tag Antibody	Cell Signaling Technology	Cat#2368;
Phospho-NF-κB p65 (Ser536) (93H1) Rabbit mAb	Cell Signaling Technology	Cat#3033
NF-κB p65 (D14E12) XP® Rabbit mAb	Cell Signaling Technology	Cat#8242
Phospho-p44/42 MAPK (Erk1/2) (Thr202/Tyr204) (D13.14.4E) XP® Rabbit mA	Cell Signaling Technology	Cat#4370
p44/42 MAPK (Erk1/2) Antibody	Cell Signaling Technology	Cat#9102
Phospho-p38 MAPK (Thr180/Tyr182) (28B10) Mouse mAb	Cell Signaling Technology	Cat#9216
p38 MAPK Antibody	Cell Signaling Technology	Cat#9212
Lamin A/C (4C11) Mouse mAb	Cell Signaling Technology	Cat#4777
IRF-3 (D83B9) Rabbit mAb	Cell Signaling Technology	Cat#4302
IL-1β (3A6) Mouse mAb	Cell Signaling Technology	Cat#12242
Mouse monoclonal anti-Rpb1 CTD (4H8)	Cell Signaling Technology	Cat#2629
Tri-Methyl-Histone H3 (Lys4) (C42D8) Rabbit mAb	Cell Signaling Technology	Cat#9751
Mouse monoclonal anti-GAPDH (3H12)	MBL	Cat#M171-3
Anti-Laminin-R antibody (A-7)	Santa Cruz Biotechnology	sc-376295
RPSA Polyclonal Antibody	ThermoFisher Scientific	PA5-86634
Anti-Phospho-Threonine Rabbit mAb	Jingjie PTM BioLab	PTM-705RM
Anti-Phospho-Tyrosine Rabbit mAb	Jingjie PTM BioLab	PTM-702
Anti-Acetyllysine Rabbit mAb	Jingjie PTM BioLab	PTM-105RM
Anti-MLKL (phospho S345) antibody	Abcam	Cat#ab196436
Anti-GSDMD antibody	Abcam	Cat#ab209845
Anti-Cleaved Caspase-3 antibody	Abcam	Cat#ab214430
Anti-HSV1 ICP8 Major DNA binding protein antibody [11E2]	Abcam	Cat#ab20194

Anti-SNF2H antibody	Abcam	Cat#ab72499
Anti-IL-6 antibody	Abcam	Cat#ab6672
APC anti-mouse/human CD11b Antibody	BioLegend	Cat#101211
FITC anti-mouse F4/80 Recombinant Antibody	BioLegend	Cat#157309
APC anti-mouse CD11c Antibody	BioLegend	Cat#117309
FITC anti-mouse I-Ek/rat RT1D Antibody	BioLegend	Cat#110205
APC anti-mouse CD49b Antibody	BioLegend	Cat#103515
FITC anti-mouse CD4 Antibody	BioLegend	Cat#100405
PE anti-mouse CD8b.2 Antibody	BioLegend	Cat#140408
APC anti-mouse CD19 Antibody	BioLegend	Cat#152409
PE anti-mouse Ly-6G/Ly-6C (Gr-1) Antibody	BioLegend	Cat#108407
DH5α	TIANGEN Biotech	Cat#CB101
BAY 11-7082	Selleck	Cat#19542-67-7
CpG ODN 1668	invivogen	tlrl-1668
Poly(I:C) LMW	invivogen	tlrl-picw
E. coli ssDNA / LyoVec™	invivogen	tlrl-ssec
cGAMP	MCE	HY-12512
G3-YSD	invivogen	tlrl-ydna
ISD	invivogen	tlrl-isdn
HSV-60	invivogen	tlrl-hsv60n
poly dI:dC	Thermo Scientific	20148E
VACV-70	invivogen	tlrl-vav70n
FuGENE HD Transfection Reagent	Promega	Cat#E2311
Lipofectamine RNAiMAX Transfection Reagent	Thermo Scientific	Cat#13778150
TRIzol Reagent	Thermo Scientific	Cat#15596018
SYBR Green Realtime PCR Master Mix	TOYOBO	Cat#QPK-201
Biotin 3’End DNA Labeling Mix	Thermo Scientific	Cat#89818
Biotin 3’End RNA Labeling Mix	Thermo Scientific	Cat#20160

Recombinant DNase I (RNase-free)	Takara	Cat#2270A
Dynabeads M-280 Streptavidin	Thermo Scientific	Cat#11205D
Dynabeads Protein G for Immunoprecipitation	Thermo Scientific	Cat#10003D
Protein A/G Magnetic Beads	Thermo Scientific	Cat#88802
ReverTra Ace qPCR RT Master Mix with gDNA Remover	TOYOBO	Cat#FSQ-301
SimpleChIP Enzymatic Chromatin IP Kit	Cell Signaling Technology	Cat#9003
Mouse IL-12/IL-23p40 Precoated ELISA Kit	Dakewe	Cat#1211232
Mouse TNF-α Precoated ELISA Kit	Dakewe	Cat#1217202
Mouse IL-1α Precoated ELISA Kit	Dakewe	Cat#1210112
Mouse IL-6 Precoated ELISA Kit	Dakewe	Cat#1210602
Mouse IL-1β Precoated ELISA Kit	Dakewe	Cat#1210122
Mouse IFN-beta DuoSet ELISA	R&D SYSTEMS	Cat#DY8234-05
Quantikine ELISA Mouse CXCL2/MIP-2 Immunoassay	R&D Systems	Cat#MM200
Quantikine ELISA Mouse CCL2/JE/MCP-1 Immunoassay	R&D Systems	Cat#MJE00B
