# Supplementary material for: Nuclear RPSA senses viral nucleic acids to promote the innate inflammatory response
Source: Nat Commun. 2023 Dec 20;14:8455. doi: 10.1038/s41467-023-43784-0 (PMC10730619; doi:10.1038/s41467-023-43784-0)
Supplement: Supplementary file 3 — Reporting Summary [file 41467_2023_43784_MOESM3_ESM.pdf]

nature portfolio

Corresponding author(s): Xuetao Cao

Last updated by author(s): Nov 5, 2023

Reporting Summary

Nature Portfolio wishes to improve the reproducibility of the work that we publish. This form provides structure for consistency and transparency in reporting. For further information on Nature Portfolio policies, see our Editorial Policies and the Editorial Policy Checklist.

Statistics

For all statistical analyses, confirm that the following items are present in the figure legend, table legend, main text, or Methods section.

n/a	Confirmed	
☐	☒	The exact sample size (*n*) for each experimental group/condition, given as a discrete number and unit of measurement
☐	☒	A statement on whether measurements were taken from distinct samples or whether the same sample was measured repeatedly
☐	☒	The statistical test(s) used AND whether they are one- or two-sided<br>*Only common tests should be described solely by name; describe more complex techniques in the Methods section.*
☒	☐	A description of all covariates tested
☒	☐	A description of any assumptions or corrections, such as tests of normality and adjustment for multiple comparisons
☐	☒	A full description of the statistical parameters including central tendency (e.g. means) or other basic estimates (e.g. regression coefficient) AND variation (e.g. standard deviation) or associated estimates of uncertainty (e.g. confidence intervals)
☐	☒	For null hypothesis testing, the test statistic (e.g. *F*, *t*, *r*) with confidence intervals, effect sizes, degrees of freedom and *P* value noted<br>*Give P values as exact values whenever suitable.*
☒	☐	For Bayesian analysis, information on the choice of priors and Markov chain Monte Carlo settings
☒	☐	For hierarchical and complex designs, identification of the appropriate level for tests and full reporting of outcomes
☒	☐	Estimates of effect sizes (e.g. Cohen's *d*, Pearson's *r*), indicating how they were calculated

Our web collection on statistics for biologists contains articles on many of the points above.

Software and code

Policy information about availability of computer code

Data collection	Sequencing data was collected by BGISEQ-500, Illumina HiSeq2000, FACS experiments were performed by BD FACS Aria II and BD LSRFortessa
Data analysis	GraphPad Prism 7, Flowjo_V10, LAS_X_Core_3.7.4, FACSDiva software

For manuscripts utilizing custom algorithms or software that are central to the research but not yet described in published literature, software must be made available to editors and reviewers. We strongly encourage code deposition in a community repository (e.g. GitHub). See the Nature Portfolio guidelines for submitting code & software for further information.

Data

Policy information about availability of data

All manuscripts must include a data availability statement. This statement should provide the following information, where applicable:

- Accession codes, unique identifiers, or web links for publicly available datasets
- A description of any restrictions on data availability
- For clinical datasets or third party data, please ensure that the statement adheres to our policy

The RNA seq/ATACseq/ChIP seq data from the present study are deposited in the National Center for Biotechnology Information's Gene Expression Omnibus under accession code GSE204895. All other study data are included in the article and/or Supplementary Information. Source data are provided with this paper.

Research involving human participants, their data, or biological material

Policy information about studies with human participants or human data. See also policy information about sex, gender (identity/presentation), and sexual orientation and race, ethnicity and racism.

Reporting on sex and gender	N/A
Reporting on race, ethnicity, or other socially relevant groupings	N/A
Population characteristics	N/A
Recruitment	N/A
Ethics oversight	N/A

Note that full information on the approval of the study protocol must also be provided in the manuscript.

Field-specific reporting

Please select the one below that is the best fit for your research. If you are not sure, read the appropriate sections before making your selection.

[x] Life sciences   [ ] Behavioural & social sciences   [ ] Ecological, evolutionary & environmental sciences

For a reference copy of the document with all sections, see nature.com/documents/nr-reporting-summary-flat.pdf

Life sciences study design

All studies must disclose on these points even when the disclosure is negative.

Sample size	No statistical methods were used to predetermine the sample size. Sample sizes were chosen based on standard practice. Group sizes for in vivo validation experiments were selected empirically based on prior knowledge of the intra-group variation of HSV-1 or IAV infection that used similar approaches. Similarly, group sizes in the in vitro viral infection experiments were selected based on prior knowledge of variation, and three biological replicates of infected cells were used (doi: 10.1126/science.aav0758., doi: 10.1038/ni.3308., doi: 10.1038/s41467-021-24724-2.).
Data exclusions	No data were excluded.
Replication	All the reported experiments were reproducible. Data reproducibility was confirmed by independent experiments. All figure legends included repeat times.
Randomization	All animal- and cell-based samples in each of the group were included and no method of randomization was applied. As the results are qualitative, the randomization was not relevant in this study.
Blinding	Blinding is not relevant to our study, as we need to know the genotypes of the cell lines and mouse strains. The phenotype change is beyond what can be affect by human bias.

Reporting for specific materials, systems and methods

We require information from authors about some types of materials, experimental systems and methods used in many studies. Here, indicate whether each material, system or method listed is relevant to your study. If you are not sure if a list item applies to your research, read the appropriate section before selecting a response.

Materials & experimental systems

n/a	Involved in the study	
[ ]	[x]	Antibodies
[ ]	[x]	Eukaryotic cell lines
[x]	[ ]	Palaeontology and archaeology
[ ]	[x]	Animals and other organisms
[x]	[ ]	Clinical data
[x]	[ ]	Dual use research of concern
[x]	[ ]	Plants

Methods

n/a	Involved in the study	
[ ]	[x]	ChIP-seq
[ ]	[x]	Flow cytometry
[x]	[ ]	MRI-based neuroimaging

Antibodies

Antibodies used	DYKDDDDK Tag Antibody Cell Signaling Technology Cat#2368;<br>Phospho-NF-κB p65 (Ser536) (93H1) Rabbit mAb, Cell Signaling Technology, Cat#3033<br>NF-κB p65 (D14E12) XP® Rabbit mAb, Cell Signaling Technology, Cat#8242<br>Phospho-p44/42 MAPK (Erk1/2) (Thr202/Tyr204) (D13.14.4E) XP® Rabbit mA, Cell Signaling Technology, Cat#4370<br>p44/42 MAPK (Erk1/2) Antibody, Cell Signaling Technology, Cat#9102<br>Phospho-p38 MAPK (Thr180/Tyr182) (28B10) Mouse mAb, Cell Signaling Technology, Cat#9216<br>p38 MAPK Antibody, Cell Signaling Technology, Cat#9212<br>Lamin A/C (4C11) Mouse mAb, Cell Signaling Technology, Cat#4777<br>IRF-3 (D83B9) Rabbit mAb, Cell Signaling Technology, Cat#4302<br>IL-1β (3A6) Mouse mAb, Cell Signaling Technology, Cat#12242<br>Mouse monoclonal anti-Rpb1 CTD (4H8), Cell Signaling Technology, Cat#2629<br>Tri-Methyl-Histone H3 (Lys4) (C42D8), Rabbit mAb Cell Signaling Technology, Cat#9751<br>Phospho-Tyrosine Mouse mAb (P-Tyr-100), Cell Signaling Technology Cat#9411<br>Mouse monoclonal anti-GAPDH (3H12), MBL, Cat#M171-3<br>Anti-Laminin-R antibody (A-7), Santa Cruz Biotechnology, sc-376295<br>RPSA Polyclonal Antibody, ThermoFisher Scientific, PA5-86634<br>Anti-Phospho-Threonine Rabbit mAb, Jingjie PTM BioLab, PTM-705RM<br>Anti-Phospho-Tyrosine Rabbit mAb, Jingjie PTM BioLab, PTM-702<br>Anti-Acetyllysine Rabbit mAb, Jingjie PTM BioLab, PTM-105RM<br>Anti-MLKL (phospho S345) antibody (EPR9515(2)), Abcam, Cat#ab196436<br>Anti-GSDMD antibody (EPR19828), Abcam, Cat#ab209845<br>Anti-Cleaved Caspase-3 antibody (EPR21032), Abcam, Cat#ab214430<br>Anti-HSV1 ICP8 Major DNA binding protein antibody [11E2] , Abcam, Cat#ab20194<br>Anti-SNF2H antibody, Abcam, Cat#ab72499<br>Anti-IL-6 antibody, Abcam, Cat#ab6672<br>Goat Anti-Mouse IgG (H+L) antibody, ZSGB-BIO, ZB-2305<br>Goat Anti-Rabbit IgG (H+L) antibody,ZSGB-BIO, ZB-2301<br>APC anti-mouse/human CD11b Antibody (M1/70), BioLegend, Cat#101211<br>FITC anti-mouse F4/80 Recombinant Antibody (QA17A29), BioLegend, Cat#157309<br>APC anti-mouse CD11c Antibody (N418), BioLegend, Cat#117309<br>FITC anti-mouse I-Ek/rat RT1D Antibody (14-4-4S), BioLegend, Cat#110205<br>APC anti-mouse CD49b Antibody (HMα2), BioLegend, Cat#103515<br>FITC anti-mouse CD4 Antibody (GK1.5), BioLegend, Cat#100405<br>PE anti-mouse CD8b.2 Antibody (53-5.8), BioLegend, Cat#140408<br>APC anti-mouse CD19 Antibody (1D3/CD19), BioLegend, Cat#152409<br>PE anti-mouse Ly-6G/Ly-6C (Gr-1) Antibody, BioLegend, Cat#108407
Validation	All antibodies are commercially available. Specific validation information and usage information can be found on their respective websites:<br>DYKDDDDK Tag Antibody, https://www.cellsignal.cn/products/primary-antibodies/dykddddk-tag-antibody-binds-to-same-epitope-as-sigma-aldrich-anti-flag-m2-antibody/2368<br>Phospho-NF-κB p65 (Ser536) (93H1) Rabbit mAb, https://www.cellsignal.cn/products/primary-antibodies/phospho-nf-kb-p65-ser536-93h1-rabbit-mab/3033<br>NF-κB p65 (D14E12) XP® Rabbit mAb, https://www.cellsignal.cn/products/primary-antibodies/nf-kb-p65-d14e12-xp-rabbit-mab/8242<br>Phospho-p44/42 MAPK (Erk1/2) (Thr202/Tyr204) (D13.14.4E) XP® Rabbit mA, https://www.cellsignal.cn/products/primary-antibodies/phospho-p44-42-mapk-erk1-2-thr202-tyr204-d13-14-4e-xp-rabbit-mab/4370<br>p44/42 MAPK (Erk1/2) Antibody, https://www.cellsignal.cn/products/primary-antibodies/p44-42-mapk-erk1-2-antibody/9102<br>Phospho-p38 MAPK (Thr180/Tyr182) (28B10) Mouse mAb, https://www.cellsignal.cn/products/primary-antibodies/phospho-p38-mapk-thr180-tyr182-28b10-mouse-mab/9216<br>p38 MAPK Antibody, https://www.cellsignal.cn/products/primary-antibodies/p38-mapk-antibody/9212<br>Lamin A/C (4C11) Mouse mAb, https://www.cellsignal.cn/products/primary-antibodies/lamin-a-c-4c11-mouse-mab/4777<br>IRF-3 (D83B9) Rabbit mAb, https://www.cellsignal.cn/products/primary-antibodies/irf-3-d83b9-rabbit-mab/4302<br>IL-1β (3A6) Mouse mAb, https://www.cellsignal.cn/products/primary-antibodies/il-1b-3a6-mouse-mab/12242<br>Mouse monoclonal anti-Rpb1 CTD (4H8), https://www.cellsignal.cn/products/primary-antibodies/rpb1-ctd-4h8-mouse-mab/2629<br>Tri-Methyl-Histone H3 (Lys4) (C42D8), Rabbit mAb, https://www.cellsignal.cn/products/primary-antibodies/tri-methyl-histone-h3-lys4-c42d8-rabbit-mab/9751<br>Phospho-Tyrosine Mouse mAb, https://www.cellsignal.cn/products/primary-antibodies/phospho-tyrosine-mouse-mab-p-tyr-100/9411<br>Mouse monoclonal anti-GAPDH (3H12), MBL, https://www.mbl-chinawide.cn/uploads/pdf/M171-3-v3.pdf<br>Anti-Laminin-R antibody (A-7), https://www.scbt.com/p/laminin-r-antibody-a-7?requestFrom=search<br>RPSA Polyclonal Antibody, https://www.thermofisher.cn/cn/zh/antibody/product/RPSA-Antibody-Polyclonal/PA5-86634<br>Anti-Phospho-Threonine Rabbit mAb, http://www.ptm-biolab.com.cn/productDetail.html?id=6386<br>Anti-Phospho-Tyrosine Rabbit mAb, Jhttp://www.ptm-biolab.com.cn/productDetail.html?id=4734<br>Anti-Acetyllysine Rabbit mAb, http://www.ptm-biolab.com.cn/productDetail.html?id=6244<br>Anti-MLKL (phospho S345) antibody, https://www.abcam.cn/products/primary-antibodies/mlkl-phospho-s345-antibody-epr95152-ab196436.html

Anti-GSDMD antibody, https://www.abcam.cn/products/primary-antibodies/gsdmd-antibody-epr19828-ab209845.html
Anti-Cleaved Caspase-3 antibody, https://www.abcam.cn/products/primary-antibodies/cleaved-caspase-3-antibody-epr21032-ab214430.html
Anti-HSV1 ICP8 Major DNA binding protein antibody [11E2] , https://www.abcam.cn/products/primary-antibodies/hsv1-icp8-major-dna-binding-protein-antibody-11e2-ab20194.html
Anti-SNF2H antibody, https://www.abcam.cn/products/primary-antibodies/snf2h-antibody-ab72499.html
Anti-IL-6 antibody, https://www.abcam.cn/products/primary-antibodies/il-6-antibody-ab6672.html
Goat Anti-Mouse IgG (H+L) antibody, http://www.zsbio.com/product/ZB-2305
Goat Anti-Rabbit IgG (H+L) antibody, http://www.zsbio.com/product/ZB-2301
APC anti-mouse/human CD11b Antibody, https://www.biolegend.com/en-us/products/apc-anti-mouse-human-cd11b-antibody-345
FITC anti-mouse F4/80 Recombinant Antibody, https://www.biolegend.com/en-us/products/fitc-anti-mouse-f480-recombinant-antibody-19715
APC anti-mouse CD11c Antibody, https://www.biolegend.com/en-us/products/apc-anti-mouse-cd11c-antibody-1813
FITC anti-mouse I-Ek/rat RT1D Antibody, https://www.biolegend.com/en-us/products/fitc-anti-mouse-i-ek-rat-rt1d-antibody-18
APC anti-mouse CD49b Antibody, https://www.biolegend.com/en-us/products/apc-anti-mouse-cd49b-antibody-12082
FITC anti-mouse CD4 Antibody, https://www.biolegend.com/en-us/products/fitc-anti-mouse-cd4-antibody-248
PE anti-mouse CD8b.2 Antibody, https://www.biolegend.com/en-us/products/pe-anti-mouse-cd8b-2-antibody-6871
APC anti-mouse CD19 Antibody, https://www.biolegend.com/en-us/products/apc-anti-mouse-cd19-antibody-13680
PE anti-mouse Ly-6G/Ly-6C (Gr-1) Antibody, https://www.biolegend.com/en-us/products/pe-anti-mouse-ly-6g-ly-6c-gr-1-antibody-460

Eukaryotic cell lines

Policy information about cell lines and Sex and Gender in Research

Cell line source(s)	RAW264.7, A549, MEF, MLE-12, HEK293T, and Vero cells were obtained from American Type Culture Collection (ATCC).
Authentication	No authentication for cell lines was performed.
Mycoplasma contamination	All cell lines were tested for mycoplasma contamination and were not contaminated.
Commonly misidentified lines (See ICLAC register)	None of these cell lines were used in this study.

Animals and other research organisms

Policy information about studies involving animals; ARRIVE guidelines recommended for reporting animal research, and Sex and Gender in Research

Laboratory animals	The transgenic mouse lines: RPSA lyzcre+ or RPSA lyz- mouse(C57BL/6. Both male and female C57BL/6 mice of 6-8 weeks. The experimental mice were bred in house in SPF condition. Control mice were littermates housed with age and sex matched for each distinct mouse strain. Mice were housed in cages with five mice per cage and kept on in a regular 12h light/12h dark cycle (lights on at 7:00 am). The temperature was 24±2 degree Celsius and humidity was 40-70%.
Wild animals	No wild animals were used in the study.
Reporting on sex	The sexually matched mice were used in all experiments.
Field-collected samples	No field-collected samples were employed in this study.
Ethics oversight	All animal protocols were approved by the Animal Care and Use Committees of the Institute of Laboratory Animal Science of Chinese Academy of Medical Sciences (ILAS-GC-2015-002).

Note that full information on the approval of the study protocol must also be provided in the manuscript.

Plants

Seed stocks	N/A
Novel plant genotypes	N/A
Authentication	N/A

ChIP-seq

Data deposition

- [x] Confirm that both raw and final processed data have been deposited in a public database such as GEO.
- [x] Confirm that you have deposited or provided access to graph files (e.g. BED files) for the called peaks.

Data access links<br>*May remain private before publication.*	ChIP seq data from the present study are deposited in the National Center for Biotechnology Information's Gene Expression Omnibus under accession code GSE204895
Files in database submission	RAW data:<br>H3K4_KO_HSV_0h_1.fq<br>H3K4_KO_HSV_0h_2.fq<br>H3K4_KO_HSV_4h_1.fq<br>H3K4_KO_HSV_4h_2.fq<br>H3K4_WT_HSV_0h_1.fq<br>H3K4_WT_HSV_0h_2.fq<br>H3K4_WT_HSV_4h_1.fq<br>H3K4_WT_HSV_4h_2.fq<br>P65_KO_HSV_0h_1.fq<br>P65_KO_HSV_0h_2.fq<br>P65_KO_HSV_4h_1.fq<br>P65_KO_HSV_4h_2.fq<br>P65_WT_HSV_0h_1.fq<br>P65_WT_HSV_0h_2.fq<br>P65_WT_HSV_4h_1.fq<br><br>Processed data:<br>H3K4_KO_HSV_0h_narrow_peaks_ChIPseeker_annotation<br>H3K4_KO_HSV_4h_narrow_peaks_ChIPseeker_annotation<br>H3K4_WT_HSV_0h_narrow_peaks_ChIPseeker_annotation<br>H3K4_WT_HSV_0h_narrow_peaks_ChIPseeker_annotation<br>P65_KO_HSV_0h_narrow_peaks_ChIPseeker_annotation<br>P65_KO_HSV_4h_narrow_peaks_ChIPseeker_annotation<br>P65_WT_HSV_0h_narrow_peaks_ChIPseeker_annotation<br>P65_WT_HSV_0h_narrow_peaks_ChIPseeker_annotation
Genome browser session<br>(e.g. UCSC)	N/A

Methodology

Replicates	Ranging from 1-3 biological replicates
Sequencing depth	10 million to 20million reads. Total number of reads: 20000000-25000000, uniquely mapped reads: 90%-95%, length of reads:100-400bp
Antibodies	see antibodies above
Peak calling parameters	After mapping reads to the reference genome, we used the MACS2 (version 2.1.0) peak calling software to identify regions of IP enrichment over background. A q-value threshold of 0.05 was used for all data sets. After peak calling, the distribution of chromosome distribution, peak width, fold enrichment, significant level and peak summit number per peak were all displayed.<br>MACS2 2.1.0 -q 0.05； --call-summits； --nomodel
Data quality	Raw data (raw reads) of fastq format were firstly processed using fastp software. In this step, clean data (clean reads) were obtained by removing reads containing adapter, reads containing ploy-N and low quality reads from raw data. At the same time, Q20, Q30 and GC content of the clean data were calculated. All the downstream analyses were based on the clean data with high quality.MACS2 scan all the genome with a specific window size and calculate the reads enrichment level. A particular number of windows were used as samples to build enrichment model and to predict fragment size. The following peak calling analysis was based on this predicted fragment size. After mapping reads to the reference genome, we used the MACS2 (version 2.1.0) peak calling software to identify regions of IP enrichment over background. A q-value threshold of 0.05 was used for all data sets. After peak calling, the distribution of chromosome distribution, peak width, fold enrichment, significant level and peak summit number per peak were all displayed.
Software	fastp 0.19.11, fastqc v0.11.5, BWA 0.7.12-r1039, deepTools(bamCorrelate bins) 1.5.9, MACS2 2.1.0, homer findMotifsGenome.pl v4.9.1, Goseq, topGO, Bioconductor(2.13) 4.10.2, KOBAS 3.0.

Flow Cytometry

Plots

Confirm that:

- [x] The axis labels state the marker and fluorochrome used (e.g. CD4-FITC).
- [x] The axis scales are clearly visible. Include numbers along axes only for bottom left plot of group (a 'group' is an analysis of identical markers).
- [x] All plots are contour plots with outliers or pseudocolor plots.
- [x] A numerical value for number of cells or percentage (with statistics) is provided.

Methodology

Sample preparation	Spleens were dissected from mice, mechanically minced, and filtered through 40uM cell strainer to single-cell suspension. RBC lysis was performed on all spleen samples. Then, stain cells with proper antibodies follow manufacturer's protocol.
Instrument	BD LSR Fortessa was used for acquisition of data
Software	BD FACS Diva, FlowJo
Cell population abundance	Only single cell sorting was performed, which was confirmed by FSC-W x FSC-H; SSC-W x SSC-H.
Gating strategy	Gating as described in the supplementary figures. In all experiments, cells were first gated on single cells based on FSC-A x SSC-A. Then, doublets were excluded twice by FSC-Ax FSC-H.

- [x] Tick this box to confirm that a figure exemplifying the gating strategy is provided in the Supplementary Information.
